# Long-range correlation properties in timing of skilled piano performance: the influence of auditory feedback and deep brain stimulation

**DOI:** 10.3389/fpsyg.2014.01030

**Published:** 2014-09-25

**Authors:** María Herrojo Ruiz, Sang Bin Hong, Holger Hennig, Eckart Altenmüller, Andrea A. Kühn

**Affiliations:** ^1^Department of Neurology, Charité-University Medicine BerlinBerlin, Germany; ^2^Department of Physics, Harvard UniversityCambridge, MA, USA; ^3^Broad Institute of Harvard and MITCambridge, MA, USA; ^4^Institute of Music Physiology and Musicians' Medicine, Hanover University of Music, Drama and MediaHanover, Germany; ^5^Cluster of Excellence NeuroCure, Charité-University Medicine BerlinBerlin, Germany

**Keywords:** timing fluctuations, music performance, long-range correlation, deep brain stimulation, Parkinson disease

## Abstract

Unintentional timing deviations during musical performance can be conceived of as timing errors. However, recent research on humanizing computer-generated music has demonstrated that timing fluctuations that exhibit long-range temporal correlations (LRTC) are preferred by human listeners. This preference can be accounted for by the ubiquitous presence of LRTC in human tapping and rhythmic performances. Interestingly, the manifestation of LRTC in tapping behavior seems to be driven in a subject-specific manner by the LRTC properties of resting-state background cortical oscillatory activity. In this framework, the current study aimed to investigate whether propagation of timing deviations during the skilled, memorized piano performance (without metronome) of 17 professional pianists exhibits LRTC and whether the structure of the correlations is influenced by the presence or absence of auditory feedback. As an additional goal, we set out to investigate the influence of altering the dynamics along the cortico-basal-ganglia-thalamo-cortical network via deep brain stimulation (DBS) on the LRTC properties of musical performance. Specifically, we investigated temporal deviations during the skilled piano performance of a non-professional pianist who was treated with subthalamic-deep brain stimulation (STN-DBS) due to severe Parkinson's disease, with predominant tremor affecting his right upper extremity. In the tremor-affected right hand, the timing fluctuations of the performance exhibited random correlations with DBS OFF. By contrast, DBS restored long-range dependency in the temporal fluctuations, corresponding with the general motor improvement on DBS. Overall, the present investigations demonstrate the presence of LRTC in skilled piano performances, indicating that unintentional temporal deviations are correlated over a wide range of time scales. This phenomenon is stable after removal of the auditory feedback, but is altered by STN-DBS, which suggests that cortico-basal ganglia-thalamocortical circuits play a role in the modulation of the serial correlations of timing fluctuations exhibited in skilled musical performance.

## Introduction

Music performance is a complicated human activity that relies on the hierarchical interplay among the sensorimotor, memory, and auditory neural systems (Altenmüller et al., 2006; Zatorre et al., 2007). Skilled music performance requires the retrieval of long sequences of events accurate in pitch and timing from memory with the aim of communicating expressive effects and emotion (Palmer, 1997; Hallam et al., 2008). Yet even professional musicians exhibit unintended fluctuations in the temporal aspect of their performance (Repp, 1999a, 2006) and, moreover, produce movement or pitch errors (Herrojo Ruiz et al., 2009, 2011; Maidhof et al., 2009). In this context, music performance can be understood as one particularly skilled example of a broader class of human performance activities which are long sequences of discrete movements. Walking, tapping to a metronome or to an internally generated beat, rhythm production, speech and writing are just a few examples of movement sequences in which the time intervals exhibit trial-to-trial variability and slower trends of variation (drift; Gilden et al., 1995; Hausdorff et al., 1995; Chen et al., 1997; Bangert and Altenmüller, 2003; Hennig et al., 2011). The ubiquitous presence of LRTC in human performance might account for the preference in human listeners for this type of temporal correlational structure during music perception (Hennig et al., 2012). Notably, however, whether temporal fluctuations in skilled music performance exhibit LRTC and whether these can be modulated by altering feedback to the performance has not been extensively studied. Answering those questions is critical to our understanding of timing properties of skilled human performance. In addition, such investigations emphasize that the analysis of correlations of time series in behavior, beyond averages of variables, is a valuable source of information.

An early attempt to model the sources of variability in a series of self-paced inter-tap-intervals (ITIs) was undertaken by Wing and Kristofferson (1973) in their two-component model. They proposed that the ITI at tap *k* is determined by two independent processes according to the following expression: ITI_*k*_ = C_*k*_ + MD_*k*_ − MD_*k* − 1_, where C_*k*_ is a timing motor command which is issued by an internal timekeeper and which is implemented by the motor system with a delay MD. The delay MD results from the ending of the previous interval *k*–1 and the beginning of current interval *k*. Both C and MD were assumed to be independent, uncorrelated white noise processes. However, further studies have established that errors in the production of time intervals are not uncorrelated, but rather exhibit long-range (persistent) correlated variation extending up to hundreds of events (Gilden et al., 1995; Hausdorff et al., 1995; Chen et al., 1997; Hennig et al., 2011). Thus, the increments of the time series are positively correlated such that a positive increase in the fluctuations of ITI over a temporal scale is most probably followed by a positive increase in the flucuations of ITI over a posterior time scale. The influential study of Gilden et al. (1995) adapted the two-component model of Wing and Kristofferson by regarding the internal timekeeper (C) as a source of 1/*f* noise. This adapted model predicted that a series of ITIs would exhibit long-range temporal correlations (LRTC) of the kind called 1/*f* noise with a positive autocorrelation structure, as further demonstrated via spectral power analysis (Gilden et al., 1995).

Alternative methods, such as the autoregressive fractionally integrated moving average (ARFIMA) modeling (Lemoine et al., 2006) or detrended fluctuation analysis (DFA, Peng et al., 1995), can also detect the structure of the correlations of serial fluctuations. DFA is a particularly popular technique for assessing the decay of temporal correlations, which might give rise to LRTC, in non-stationary physiological and behavioral time series (Peng et al., 1995). DFA (opearting in the time domain) and spectral analysis (operating in the frequency domain) can be considered as complementary approaches to estimate the scaling exponents of long-term correlations in stationary stochastic signals (Heneghan and McDarby, 2000).

Recent work has shown that the manifestation of LRTC in tapping behavior seems to be driven in a subject-specific manner by the LRTC properties of resting-state background cortical oscillatory activity (Smit et al., 2013). LRTCs are indeed ubiquitous across different temporal and spatial scales of neuronal activity (Linkenkaer-Hansen et al., 2001, 2004; Plenz and Chialvo, 2009; Palva et al., 2013). However, the scaling exponents can be modulated via sensory stimulation and in different neurological or psychiatrical disorders (Linkenkaer-Hansen et al., 2004; Montez et al., 2009; Nikulin et al., 2012). In particular, somatosensory stimuli can attenuate the scaling exponents in brain activity (Linkenkaer-Hansen et al., 2004), reflecting a change in the underlying oscillatory dynamics.

In this study, our first goal was to investigate whether the statistical structure of the serial correlations during overlearned piano performance (without metronome) of skilled pianists is affected by the presence or absence of auditory feedback. According to the ideomotor theory of action control (e.g., Prinz, 1997), the motor action is bound with the sensory effects it produces. This link arises after frequent performance of the specific action and the learning of the sensory effects associated with it (Elsner and Hommel, 2001) and leads to the strong auditory-motor coupling observed in musicians (Bangert and Altenmüller, 2003; Drost et al., 2005a,b; Zatorre et al., 2007). In line with this evidence, studies have demonstrated that once the pieces are learned, their retrieval from memory is independent of the presence or absence of auditory feedback (Repp, 1999b; Finney and Palmer, 2003). Additionally, auditory feedback does not influence the neural mechanisms underlying error prediction (Herrojo Ruiz et al., 2009). Accordingly, we reasoned that at an automatic stage of performance after intensive training, auditory feedback would not influence the dynamics of the temporal fluctuations. To test our hypothesis, we evaluated, the time series of isochronous inter-onset-intervals (IOI, time between note onsets of two subsequent notes) during the performance of piano pieces by Bach with spectral analysis and DFA. The stimulus material consisted of isochronous IOIs and note durations.

As an additional goal, we set out to investigate the influence of subthalamic deep brain stimulation (STN-DBS) on the strength of LRTC during skilled piano performances. Deep brain stimulation (DBS) is a well-established treatment for patients with severe movement disorders (Starr et al., 1998). One of the possible mechanisms of DBS is the disruption of the abnormal pattern of neuronal activity within the cortico-basal ganglia- thalamo-cortical network in those patients via high-frequency stimulation (typically at 130–180 Hz; see review in McIntyre and Hahn, 2010). Dynamical properties of the cortico-basal-ganglia-thalamo-cortical network can be assessed experimentally via alterations in the DBS parameters settings (Eusebio et al., 2008). Accordingly, assessment of the influence of STN-DBS on the LRTC properties of piano performance might provide evidence regarding the direct involvement of the cortico-basal ganglia circuits in the modulation of temporal fluctuations during performance.

Recently, it has been suggested that thalamic DBS in essential tremor may release the dynamics of the cortico-striatopallidal-thamalocortical loops and enhance the strength of LRTC in cortical beta oscillations (Hohlefeld et al., 2013). Against this background, we hypothesized that STN-DBS would interact with the scaling exponents characterizing the temporal correlations during performance. To test our hypothesis, we investigated the time series of temporal deviations during skilled piano performance of a pianist who was treated with STN-DBS due to Parkinson's disease (PD), with predominant tremor affecting his right upper extremity. PD is a particularly interesting case study considering that previous investigations have reported in this group of patients uncorrelated temporal intervals during production of rhythmic movements, such as in gait (Hausdorff, 2009). The gradual return of the fluctuations to the healthy LRTC range can be triggered by dopaminergic medication and rhythmic auditory cuing and, thus, parallels improvements in motor symptoms (Cruz et al., 2009; Hove et al., 2012). Accordingly, in the present work we placed special emphasis on detecting transitions from uncorrelated to persistent, long-range correlated behavior during DBS.

## Materials and methods

### Participants in study 1

The performance data of 17 healthy right-handed pianists from Herrojo Ruiz et al. (2009; eight females, age range 20–29 years, mean 22 years) were reanalyzed for the investigation of LRTC of the IOI (ms). All participants were students at or had graduated from the University of Music and Drama of Hanover. They gave informed written consent to participate in the study, which was approved by the ethics committee of the University of Music and Drama of Hanover.

### Stimulus materials for study 1

**Figure 1:**
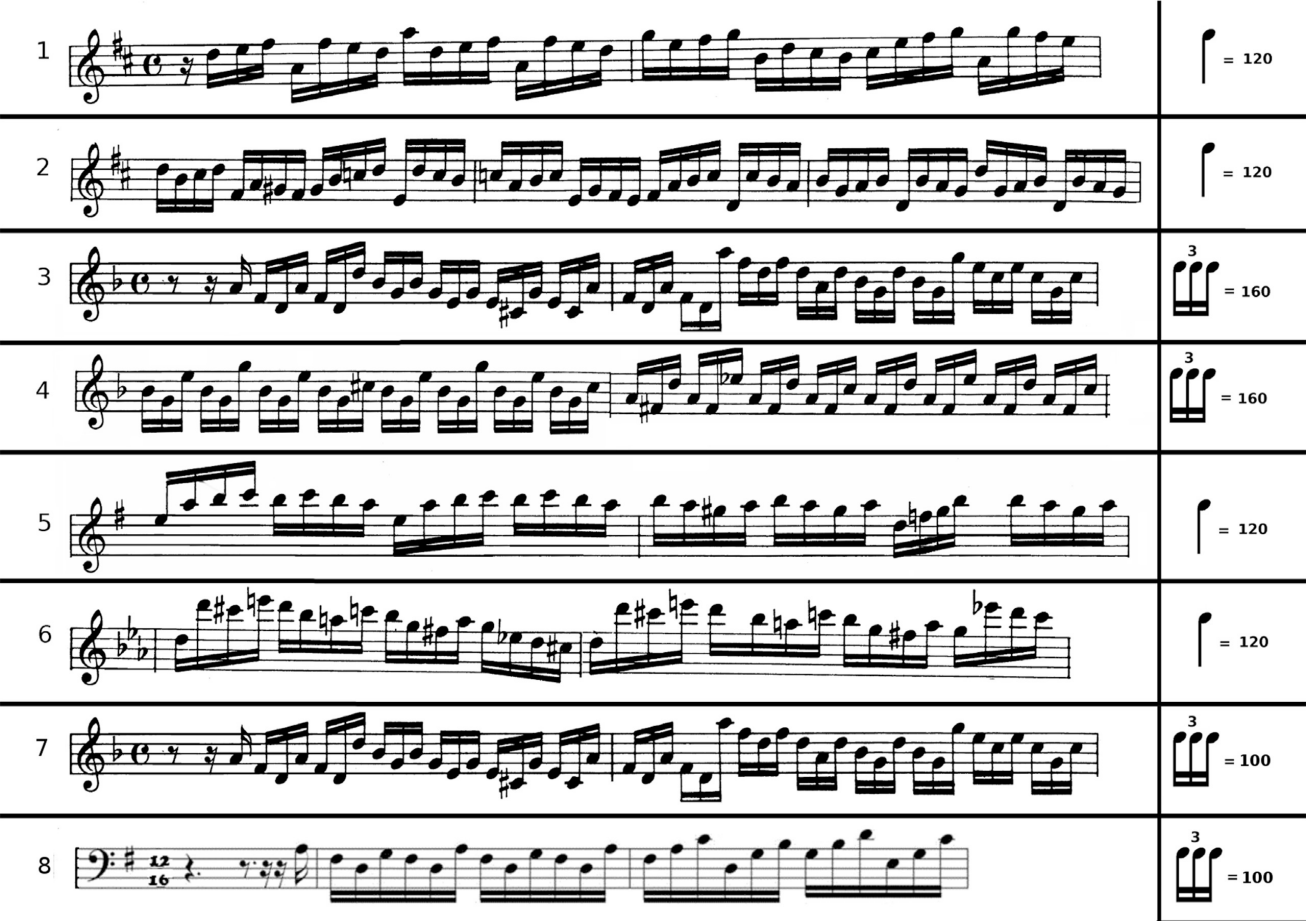
**Examples of musical stimuli**. The first bars of the six musical sequences for study 1 are illustrated. Pieces 1 and 2 were adapted from the *Prelude V* of the *Well-Tempered Clavier* (Part 1) of Bach, pieces 3 and 4 were adapted from the *Prelude VI* and piece 5 from the *Prelude X*. The sixth sequence was adapted from the *Piano Sonata No. 52 in E Flat Major* of Haydn. The *tempi* as were given in the experiment are indicated: metronome 120 for quarter note and 160 for triplet of sixteenth notes. In all cases, the IOI was 125 ms. For study 2 the musical stimuli consisted of one piece (piece 3 from study 1, denoted here by label 7) for the right hand and one piece adapted from the *Gigue, French Suite V in G major* BWV 816 by J. S. Bach, for the left hand (denoted by label 8). The *tempo* for playing the musical sequences for study 2 was: metronome 100 for triplet of sixteenth notes. Thus, the IOI was 200 ms.

The stimuli were six sequences extracted from the right-hand parts of the *Preludes V, VI*, and *X* of The Well-Tempered Clavier (Part 1) by J. S. Bach and the Piano Sonata No. 52 in E Flat Major by J. Haydn. These pieces were chosen because their parts for the right hand contain mostly one voice consisting of notes of the same value (duration), sixteenth-notes, which made our stimulus material homogeneous. The number of notes per sequence was around 200. The tempo for each piece was selected so that the ideal IOI was 125 ms (8 tones per second) in all cases. Most pieces were familiar to all pianists. However, they were instructed to rehearse and memorize them before the experimental session. During the rehearsing sessions, the given tempi were paced by a metronome. More details of the stimuli can be obtained in Figure 1 and in Herrojo Ruiz et al. (2009).

### Experimental design for study 1

Participants were seated at a digital piano (Wersi Digital Piano CT2) in a light-dimmed room. They sat comfortably in an arm-chair with the left forearm resting on the left armrest of the chair. The right forearm was supported by a movable armrest attached to a sled-type device that allowed effortless movements of the right hand along the keyboard of the piano. Instructions were displayed on a TV monitor (angle 4°) located above the piano. Before the experiment, we tested whether each pianist was able to perform all musical sequences according to the score and in the desired tempo. They were instructed to perform the pieces each time from beginning to end without stopping to correct errors. Playing the correct notes and maintaining accurate timing were stressed.

The experimental design consisted of two conditions (audiomotor, motor) comprising 60 trials (around 12000 notes). In the audiomotor condition pianists could listen to the auditory feedback of the notes played. In the motor condition, there was no auditory feedback and pianists played on a mute piano. In each condition, the 60 trials were randomly selected out of the 6 stimulus materials. The task was to play the musical stimuli 1–6 from memory without the music score. The specifications of each trial were as follows: the pianists pressed the left pedal when they were ready for a trial. After a silent time interval of 500 ± 500 ms randomized, the first two bars of the music score were presented visually on the monitor for 4000 ms to indicate which of the 6 sequences had to be played. To remind the participant of the instructed peformance tempo for each musical piece, we used a synchronization–continuation paradigm. After 2500 ms of the visual cue, the metronome started and paced the tempo corresponding to the piece for 1500 ms and then faded out. After the last metronome beat, the visual cue vanished. Participants were instructed not to play while the music score was displayed on the screen, but to start playing after a green ellipse appeared on the monitor (100 ms after the vanishing of metronome and visual cue with the score). Performance was recorded as MIDI (Music instruments digital interface) files using a standard MIDI sequencer program. The timing accuracy of the MIDI recordings was below 1 ms (tested using sequences of metronome clicks with fixed inter-click intervals.).

#### Participant and surgery in study 2

We conducted a new experiment with a male pianist that had undergone DBS therapy 12 months before for treatment of idiopathic Parkinson's Disease (Age 46, onset age 38). The patient suffered from tremor-dominant PD on his right hand, bradykinesia and rigidity. No levodopa-induced dyskinesias or motor fluctuations were present. PD motor symptoms at the time of the study were rated by an experienced movement disorder's specialist using the Unified Parkinson's Disease Rating Scale III (UPDRS-III: 11/108 ON DBS, OFF medication; 18/108 OFF DBS, OFF medication). Chronic DBS at stimulation parameters (R1-, 1.7 V, 130 Hz, 60 μs; L1–, 5 V., 130 Hz, 60 μs) was successful to significantly suppress tremor as well as ameliorate bradykinesia and rigidity without the need of further dopaminergic medication (mean decrease of 61% in the UPDRS-III ON compared to OFF DBS). The patient was not a professional pianist but was highly trained (cummulative training time > 10,000 h). Major cognitive disturbances were ruled out prior to surgery by appropriate neuropsychological [DemTect cognitive screening test to detect mild cognitive impairments and early dementia = 18 (best score)]. The patient gave informed written consent to participate in the study, which was approved by the ethics committee of the Charité—University of Medicine, Berlin.

**Figure 2:**
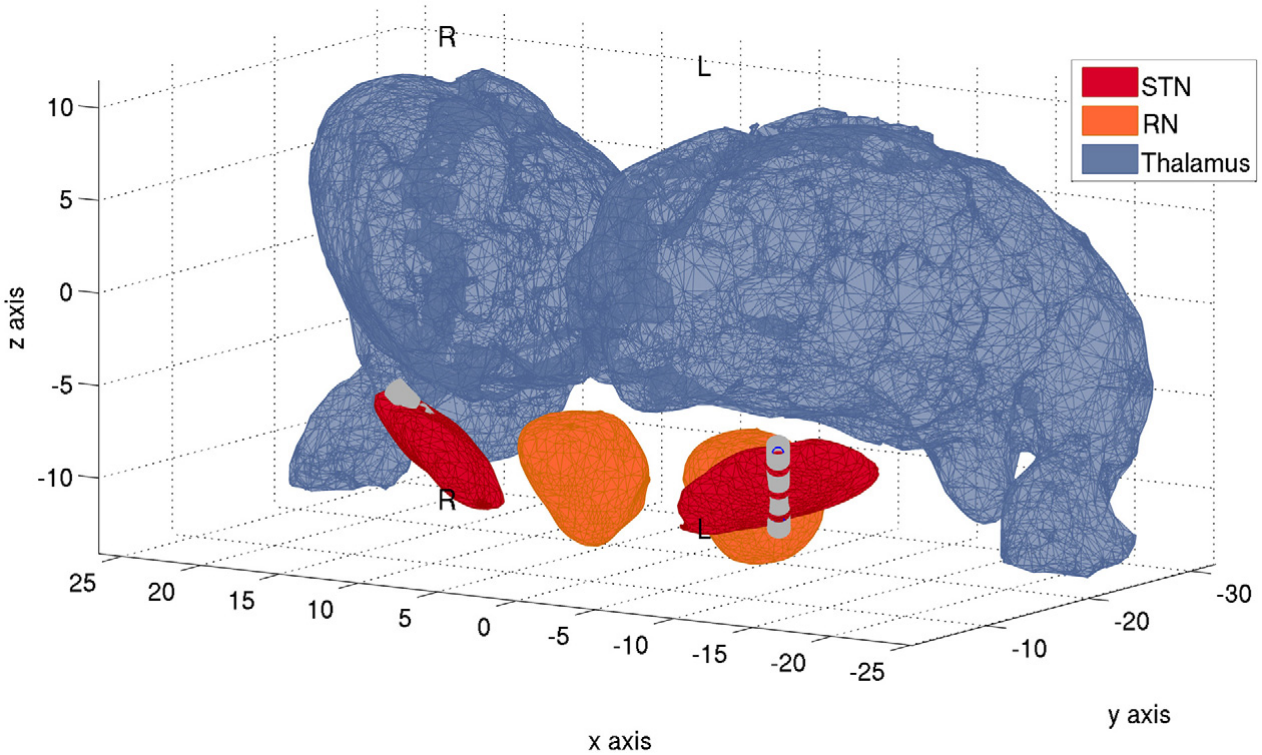
**Localization of electrodes within the STN**. Anatomical localizations of the quadripolar Medtronic DBS leads bilaterally, with 4 electrode contacts each. Electrode contacts are denoted by the gray cilinders. Reconstruction of electrode trajectory was performed as in Horn et al. (2013) and after normalization of pre-surgical MR-images into standard space. More ventral electrode contacts (0) lie toward the negative z-axis, whereas more dorsal contact electrodes (3) lie toward the direction of positive z-axis. In the left STN, contacts 1 and 2 lie within the STN, contact 0 intersects significantly with it and the most dorsal contact 3 only intersects minimally with it. In the right STN, contacts 0-1-2 lie within the boundaries of the STN, whereas the most dorsal contact 3 intersects minimally with it. Plot performed with MATLAB® function inpolyedron.

DBS electrodes were targeted bilaterally in the dorsolateral “motor” portion of the STN. A model 3389 macroelectrode was used, with four platinum-iridium cylindrical surfaces (1.27 mm in diameter and 1.5 mm in length) along a ventral to dorsal axis. Electrode placement was verified on the post-operative MRI according to the semi- automated approach described in Horn et al. (2013). Post-operative MRIs confirmed that contacts 0-1-2 were located in the left STN and also right STN, respectively, whereas the uppermost contacts 3 only intersected minimally with the STN (Figure 2). The participant gave informed written consent to participate in the study, which was approved by the local ethics committee.

#### Stimulus materials for study 2

Due to the tremor on the dominant right hand of the pianist with PD, we aimed to assess his piano performance separately for each hand. For performance with the right hand we selected one of the music pieces used in study 1, the sequence extracted from the right-hand part of the *Prelude VI* in D minor of The *Well-Tempered Clavier* (Part 1) by J. S. Bach. For performance with the left hand we selected and modified a sequence from the left-hand part of the *Gigue, French Suite V in G major* BWV 816 by J. S. Bach (Figure 1). This part was adapted by filling in some triplets with missing notes from implied harmonies, or from transposing right hand parts if they were suitable. In filling in missing notes, the note transitions were constrained not to be larger than the largest one in the right-hand part of the *Prelude VI* (a 12th). Both sequences had a similar rate of harmonic progression (around 1 chord change per two triplets) and similar harmonic complexity, since both pieces went only as far as dominants of diatonic chords. Thus, the chosen pieces contained triplets, and the single pitches had typically the same value (duration), sixteenth-notes, which make our stimulus material homogeneous (see details in Figure 1). All stimuli constituted complete musical phrases and had 200 and 205 notes per sequence, respectively. The tempo for each piece was selected so that the IOI was 200 ms (between each note within 1 triplet of semiquavers/s) in sequence one for the right hand, and 250 ms (between each note within 1 triplet of semiquavers/s) in sequence two for the left hand. The pianist was instructed to rehearse and memorize the pieces with the given tempo before the experimental session. He reported to have rehearsed, however, both pieces at the same tempo of one keystroke every 200 ms (Figure 1). Note that in this study the stimulus material was limited to one sequence per hand because we tested both DBS ON and OFF conditions, which already lengthened the experiment. No recordings without auditory feedback could be performed either due to time constrains.

#### Experimental design for study 2

The patient was seated at a digital piano (Yamaha Arius YDP-161) in a light-dimmed room. He sat comfortably in an arm-chair with the forearm contralateral to the performance side resting on the armrest of the chair. Instructions for the patient were the same as those used in study 1 (see above). Before the experiment, we tested whether the patient was able to perform both musical sequences according to the score and in the desired (rehearsed) tempo while being on the clinical DBS settings.

The experimental design consisted of four conditions comprising four trials for each sequence type (performed with either the left or right hand). The four experimental conditions corresponded with different randomized stimulation settings as follows: (1) DBS ON (see above for clinical parameters), (2) DBS OFF, (3) DBS ON, (4) DBS OFF. Thus, stimulation conditions DBS ON and OFF were repeated twice during the experiment. Note that the MIDI recordings were performed without dopaminergic medication as consistent with the post-surgery improvement of the patient's motor symptoms. Following changes in stimulation parameters we waited for at least 10 min before the next experimental test to enable stabilization of the DBS settings. In each stimulation condition, we inferred the clinical *fine-motor control* state of the patient by assessing the standard deviation of the IOI during performance with the tremor-affected right hand. In each condition and for each sequence type the task was to play with the corresponding hand the musical stimulus from memory without the music score, while listening to the auditory feedback of the notes played. The specifications of each trial were as indicated in study 1, with the difference that the metronome cues presented prior to each trial were consistent with the tempo rehearsed for the left and right hand parts, 200 ms IOI. As in study 1, performance was recorded as MIDI files and the MIDI timing accuracy was below 1 ms.

### Data analysis

#### Performance analysis for studies 1 and 2

From the MIDI files, we extracted information regarding the time between the onset of consecutive notes (IOI, ms). Timing performance for each playing condition was characterized by the mean IOI (mIOI) and the mean standard deviation of IOI (sdIOI). The latter parameter is related to the temporal unevenness of IOI. The (mIOI) provided an indication of how well the pianists and the patient adjusted to the given tempi.

For each sequence type and participant separately, we extracted the time series of IOI for each trial and concatenated trials from different renditions of the same sequence type (*N* = 10 trials for each sequence type in study 1; *N* = 4 in study 2). Although for the analysis of LRTC continuous time series are typically used, concatenation of physiological or behavioral time series has also been previously used (Hohlefeld et al., 2012; See also below in this section). It could be argued that the concatenation procedure is not adequate for a synchronization-continuation paradigm as ours, in which the tempo was paced prior to the beginning of each trial. The tempo cues could induce a resetting of timing processes at the beginning of each trial, whereas timing regularity and adjustment to the tempo could degrade across time as performance in the trial unfolds. Accordingly, here we set a strict criterion to accept the concatenation of trials: exclusively musical sequences that were characterized by a constant average tempo and timing regularity (sdIOI) at the beginning, middle and end of the trial (assessed in segments of 40 keystrokes, respectively) were accepted for further analyses of the serial correlations in concatenated time series. We predicted that the instructed tempo should be internalized in the pianists as a result of the required intensive training of the music patterns with a metronome prior to the recording session. Consequently, we expected that performance of at least some sequence types would fulfill our strict concatentation criterion.

The measure of the LRTC in the concatenated time series of IOIs was assessed with two complementary methods, DFA and analysis of the power spectral density (PSD). The time series of IOIs spanned around 2000 keystrokes per sequence type in study 1 and 800 keystrokes per musical sequence in study 2. As in previous studies (Gilden et al., 1995; Hennig et al., 2011; Smit et al., 2013) events with too short (<50 ms) or two large (>800 ms) IOI were discarded because they are related to action slips (double keypress) or memory slips (event retrieved too late).

The DFA was applied step-wise as follows:

1. A cumulative sum (integral) of the time series shifted by the mean was calculated and then segmented into 20 non-overlapping windows of equal size τ ranging from 5 to 200 keystrokes (approximately the length of each musical sequence) on a logarithmic scale. The segmentation of the time series excluded discontinuities in the data caused by the concatenation of trials corresponding to different renditions of the same musical sequence.
2. In each segmentation (without discontinuities), the integrated data *Y* (*t*) was locally fit by the least-squares method to obtain the polynomial (linear: first-order DFA) *Y*_τ_ (*t*). Then, *Y* (*t*) was linearly detrended in each interval τ by subtracting the local trend *Y*_τ_ (*t*) and the mean-squared residual (fluctuation) *F*_τ_ (*t*) was evaluated:
  (1) $$F^{2}\left(\tau \right)=\frac{1}{N}\sum_{t\;=\;1}^{N}{\left[Y\left(t\right)-Y_{\tau }\left(t\right)\right]}^{2},$$

where *N* is the total number of IOI values in the time series. The relation between variables *F*(_τ_) and the window size τ on a double log–log plot may be linear *F* (τ) ∝ τ^α^, reflecting power-law scaling behavior, which is an indication of self-similarity properties. The slope of the line is the scaling exponent α. A scaling exponent 0.5 < α < 1 indicates that LRTC are present in the signal, whereas = 0.5 reflects uncorrelated processes (white noise).

Preprocessing of the data for spectral analysis consisted of the following steps: (i) substracting the mean at each value of the time series, (ii) tapering the signal at its edges by applying a Hanning window, and (iii) linearly detrending (following Torre and Wagenmakers, 2009). After these preprocessing steps, we applied the Fast Fourier Transform to the time series to obtain the PSD. The slope β of the PSD in the log–log representation was extracted for the range of frequencies associated with long-range correlations (following Gilden et al., 1995), 1/200 < *f* < 0.02/*T*_0_, where *T*_0_ was 0.125 and 0.200 s for studies 1 and 2, respectively. We used 1/200 as lower frequency cutoff for the power law fit, because the length of each concatenated sequence was around 200 keystrokes (see above DFA). Of note, for stationary signals in the regime of LRTC the power-spectrum exponent β is related to the DFA scaling exponent approximately as β = 2α − 1 (Buldyrev et al., 1995).

#### Simulation of time series of know scaling exponents

Notably, estimators for 1/*f*^β^ noise and LRTC in experimental time series, such as PSD and DFA, inevitably have intrinsic errors and bias due to the finite length of the sequences. The bias is the difference between the estimated exponent and the true exponent, whereas the intrinsic error is the standard deviation of the estimator (see below and Pilgram and Kaplan, 1998). The intrinsic errors are fundamentally different and larger than the errors obtained by a least square fit in a log–log plot. Thus, a “good” fit in a log–log plot can provide a too optimistic estimate of the actual error involved. In Pilgram and Kaplan (1998) the intrinsic error and bias were estimated for PSD (and similarly for DFA) in the following way. A number of *m* = 50 realizations of a process with known exponent β were generated and then for each of them an estimation of the measured exponent β*_m_* was calculated using PSD. From the distribution of measured exponents β*_m_*, the bias (β- mean[β*_m_*]) and intrinsic error (SD[β*_m_*]) in the estimation of the true exponent β were calculated. Here we proceeded in a similar fashion but with time series of length *N* = 2000 or 800, corresponding to our experimental data, that resulted from concatenation of segments of 200 keystrokes. Accordingly, we generated for a known exponent realizations of a fractional Brownian motion process (non-stationary, fBm) of length 200 and concatenated them in sets of 10 or 4 to produce *m* = 500 realizations of total length 2000 or 800, respectively. The realizations of fBm were generated in MATLAB with function wfbm (Figure S1A), which produces a wavelet-based synthesis for the fBm process based on the algorithm proposed by Abry and Sellan (1996). A distribution of 500 fBm realizations was computed for each known Hurst exponent in the range 0.5:0.01:1 (see details of the Hurst exponent that characterizes fractal systems in Mandelbrot, 1982). Next, we generated with the increments of the fBm process *X*(*t*) a fractional Gaussian noise (fGn, Figure S1B) process *Y*(*t*) as: [*Y*(*t*) = *X*(*t* + 1) − *X*(*t*)]. We then applied DFA and PSD (here *T*_0_ = 1 s) for each of the m realizations of *Y*(*t*) to estimate α and β exponents, respectively, as detailed above, and assessed the bias and intrinsic error in the estimation of these exponents. Note that the estimation of the α and β exponents based on the realizations of the fGn (differentiation of fBn) process and in the LRTC regime relates to the known Hurst exponent approximately according to relations, α = H and β = 2H-1 (Buldyrev et al., 1995). Thus, with this procedure we were able to quantitatively assess and correct the specific bias introduced in our DFA and PSD calculations by the finite sequence length and, additionally, by the concatenation of shorter segments (here we compared continuous and concatenated time series, Figures S1C–S1F).

The results of the simulations revealed that for concatenated time series of total length *N* = 2000 the DFA-based bias in the estimation of the true exponent α decreases mononically from 0.015 to 0.005 for increasing H (Figure S1C). The PSD-based estimation bias of the scaling exponent β shows an U-shaped modulation with minimum bias (0.01) at H within 0.55–0.60 (Figure S1E). The intrinsic error increases monotonically from 0.025 (at *H* > 0.5) in DFA and remains around 0.06 in PSD. In the case of concatenated time series of length *N* = 800 the bias oscillates around 0.01 for DFA, whereas for PSD the bias exhibits an U-shaped modulation with minimum values of 0.02 at H within 0.55–0.60. The intrinsic error increases from 0.04 in DFA and stays around 0.07 in PSD. As observed in Figure S1, the bias in the estimation of the scaling exponents is typically larger in concatenated relative to continuous time series but the intrinsic error is similar.

Throughout this manuscript the scaling exponents α and β will be provided *after* subtraction of the corresponding bias and accompanied by estimation of the intrinsic error.

### Statistics

In study 1, statistical differences between conditions were assessed by means of a non-parametric paired permutation test (Good, 2005) across subjects (*N* = 17), with a total of 5000 random permutations. We used as test statistic the difference in sample means. The *p*-values were computed as the frequencies that the replications of the test statistic had absolute values greater than or equal to the experimental difference. Following this approach we evaluated the following variables: (i) mIOI compared between the first and middle 40 keystrokes of within-trial performance (averaged across trials for each sequence type), and also mIOI compared between the first and last 40 keystrokes of within-trial performance; (ii) similarly for sdIOI. These tests aimed to evaluate for each sequence type the stability of timing parameters during the trials and to accept or reject concatenation of data from different renditions of the same music piece. In addition, we assessed (iii) the effect of auditory feedback on timing performance (mIOI and sdIOI) and strength of LRTC (scaling exponents from DFA and PSD analyses). To this aim, conditions with and without auditory feedback were contrasted.

In study 2, a two-factorial analysis of the timing parameters mIOI and sdIOI with factors Hand (L/R) and DBS (ON, OFF) was performed by means of paired synchronized permutations across trials (8 trials from two DBS ON conditions and 8 trials from two DBS OFF conditions) with the full permutation distribution with 2^8^ values (Good, 2005; Basso et al., 2007). Synchronized permutations are based on the non-parametric pairwise permutation test and are recommended to obtain exact tests of hypotheses when two factors are involved. They are generated, for instance, by exchanging elements between rows in one column and duplicating these exchanges in all other columns (testing for effects of Factor A), or permuting indices of samples between columns and simultaneously for each row (testing for effects of Factor B). Synchronized permutations provide a clear separation of main effects and interactions and allow for non-parametric exact testing on each factor. Furthermore, as *post-hoc* analysis we assessed statistical differences in the timing parameters between stimulation conditions (ON, OFF) by means of a paired permutation test across trials (*N* = 8, total number of permutations 2^8^).

Effects were considered significant if *p* < 0.05. Multiple comparisons were corrected by controlling the false discovery rate (FDR) at level *q* = 0.05 by means of an adaptive two-stage linear step-up procedure (Benjamini et al., 2006). FDR control was performed whenever several hypotheses were evaluated under the same test statistic (for instance, effect of auditory feedback on the average IOI tested for sequences 1–6 separately: 6 multiple comparisons), but not when several different test statistics were assessed (for instance, effect of DBS on the sdIOI and, separately, on the scaling exponents). The corrected threshold *p*-value obtained from the FDR control procedure, termed pth, is given when multiple comparisons were performed.

Finally, in addition to the results of the paired permutation tests for the difference between two sample means, we report a non-parametric effect size estimator, PS_dep_, following Grissom and Kim (2012). PS_dep_ is the probability that in a randomly sampled pair of values (one matched pair) the value from Condition B (which for instance has larger values) will be greater than the value from Condition A. We can proceed as follows: for two samples of length *N*, we first compute the difference between each of the *N* pairs of values from both samples, then we count the number of positive difference scores *N*+. The probability of greater values in sample B relative to A is PS_dep_ = *N+/N*. If there are ties (zero difference), we reduce the denominator *N* by the number of ties N0 [PS_dep_ = *N+*/(*N*–*N*0)]. A non-parametric estimation of effect size like PS_dep_ is more adequate when using non-parametric tests than reporting parametric effect size estimates such as Cohen's *d*, particularly because parametric effect size estimates are affected by deviations from normality and heterogeneity of variances.

## Results

### Long-range temporal correlations in sequences of musical performance

**Figure 3:**
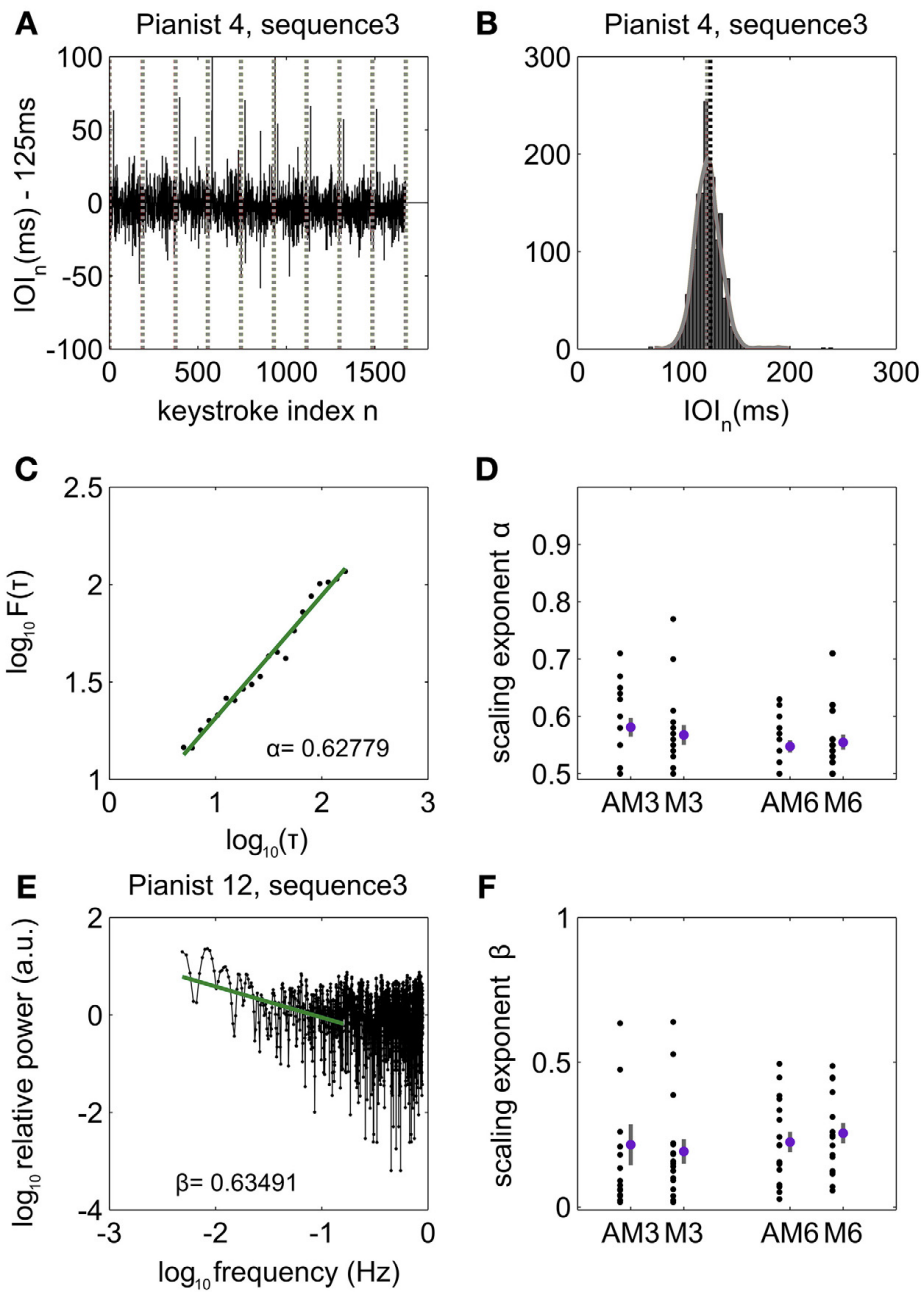
**Long-range temporal correlations during piano performance. (A)** Representative time series of inter-onset-interval (IOI, ms) values in pianist #4, during performance of sequence type 3. Vertical lines denote boundaries between trials corresponding to renditions of the same sequence type. The IOI fluctuates around the nominal IOI of 125 ms with a mean of −2.19 ms, indicating that on average the pianist played very closely to the rehearsed tempo. **(B)** The probability density function (pdf) of the time series of IOI in all patients and conditions was non-Gaussian and right skewed in 51% of all cases (tested for each patient, sequence type and auditory feedback condition separately: chi-square goodness-of-fit test rejected the null hypothesis of the distribution being a normal distribution, *p* < *p*th = 0.0001), whereas in 49% of all cases the null hypothesis of Gaussian distribution could not be rejected at the 0.05 level. Here the histogram and fitted pdf are displayed for the IOI values of pianist #4 performing sequence type 3. **(C)** log–log plot showing power-law scaling behavior for the fluctuation function *F* (τ) with increasing time scales τ spanning 5–200 keystrokes. *F* (τ) is computed from the variance of the detrended time signal. The scaling exponent α represents the slope of the linar fit. Representative patient #6 playing musical piece 3. **(D)** Distribution of the first-order DFA scaling exponents across all pianists (black dots) during audiomotor (AM) and motor (M) conditions and playing musical sequence 3 and 6. The values of the scaling exponents are displayed after subtraction of the bias introduced by DFA in the estimation of the degree of LRTC. The mean and standard error of the distribution of bias-corrected scaling exponents for each condition AM3, M3, AM6, M6 are plotted right to the distribution of the values from all pianists. **(E)** Spectral power density plotted against the log base 10 of the frequency > 10/*N*, with N being the data length (~2000 keystrokes). Representative case for pianist 12 playing sequence 3. The segment between 10/*N* < *f* < 0.02/*T*_0_, with *T*_0_ = 0.200 s (instructed IOI), corresponded to power-law 1/*f*^β^ scaling behavior and is represented here by the green line (following Gilden et al., 1995). The best fit line to the PSD in this range had exponent β = 0.63491. Note that PSD data for higher frequencies above 0.02/*T*_0_ (keystroke interval < 6) corresponds to short-range interval-to-interval variability and typically shows uncorrelated (as in this case, β ~ 0) or anti-correlated fluctuations. **(F)** Distribution of the PSD bias-corrected scaling exponents β displayed as single-values across all pianists (black dots) and as mean and SE (pink dot and gray bar, respectively) for conditions AM3, M3, AM6, and M6.

Here we describe the results of the analysis of the IOI in keystrokes correct in pitch. Unless otherwise stated, when average values are provided they are accompanied by the standard error of the mean (SE). Detailed performance data concerning error rates can be found in Herrojo Ruiz et al. (2009). Pianists played each sequence type at the instructed and rehearsed tempo corresponding to an IOI of 125 ms [average IOI not significantly (n.s.) different from 125 ms, *p* > *p*th = 0.01 after control of FDR, n.s. in all cases except for sequence type 3, see below], and regardless of the presence (audiomotor = AM) or absence (motor = M) of auditory feedback (n.s. difference between AM and M in the average IOI values, *p* > *p*th = 0.01). Exclusively for sequence type 3 pianists played at an average tempo significantly faster than the nominal tempo [117.1 (1.79) ms and 118.9 (2.21) ms for AM and M, respectively; *p* < *p*th = 0.01 and PS_dep_ = 0.8824 and 0.6471, respectively]. For sequence types 3 and 6, the sdIOI and mIOI were not significantly different between the middle or end of the trial relative to the beginning of the trial, and either between the first and last rendition of that sequence type (*p* > *p*th = 0.019 in all cases, Figure 3A). By contrast, pianists played musical pieces 1–2 and 4–5 with significantly larger temporal accuracy (smaller sdIOI) by the end of the sequence rendition, and at the last trial (*p* < *p*th = 0.019, PS_dep_ in range 0.62–0.85 for musical pieces 1, 2, 4, 5), which could be associated with a performance-related learning effect.

Accordingly, in the following we present DFA and PSD results corresponding with performance of musical pieces 3 and 6 and in concatenated time series of up to a length of 2000 keystrokes. Of note, the null hypothesis that the variable IOI is a random sample from a normal distribution with mean and variance estimated from the time series of IOIs was rejected in 51% of all cases (tested for each pianist, sequence type and auditory feedback condition separately: chi-square goodness-of-fit, *p* < *p*th = 0.0001). The distributions of IOIs were typically right skewed (Figure 3B).

First-order DFA demonstrated for the majority of the pianists (71–88% of all cases) the presence of LRTC in the fluctuations of IOI corresponding with scaling exponents significantly larger than 0.5, even after correcting for the bias in the estimation of the exponents (*p* < *p*th = 0.0002, PS_dep_ in range 0.71–0.88 in all cases: SEQ3 and SEQ6, and AM and M; Figures 3C,D). The LRTC extended from 5 to 200 keystrokes, and the scaling exponents were on average 0.577 (0.0232) and 0.551 (0.0134) for sequences 3 and 6, respectively, and in AM; scaling exponents in M were 0.574 (0.0186) and 0.554 (0.0177). The average intrinsic error in the estimation of DFA scaling exponents was around 0.01 for both conditions and sequence types. Scaling exponents from sequences 3 and 6 were not significantly correlated (*p* > 0.05, n.s.). Furthermore, when pianists played with and without auditory feedback the scaling exponents were similar on average (*p* > *p*th = 0, n.s. Note that a threshold *p*-value at 0 obtained after control of FDR implies that none of the multiple comparisons can be rejected, since they typically lie in the range *p* >> 0.05), demonstrating that auditory feedback during automatic overlearned performance does not modify the structure of the temporal correlations of the intervals between consecutive keystrokes.

The fluctuations in the series of IOIs exhibited 1/*f*^β^ power-law behavior for all pianists with average exponents β 0.21 (0.070) for SEQ3 and 0.22 (0.035) for SEQ6 in AM (example in Figure 3E); β was on average 0.19 (0.042) for SEQ3 and 0.25 (0.035) for SEQ6 in M. The intrinsic error in the estimations was around 0.06. Exponents in both conditions were not significantly different (*p* > *p*th = 0, n.s.; Figure 3F). This confirms that the statistical structure of the serial correlations exhibited during overlearned piano performance in skilled pianists is not affected by the presence or absence of auditory feedback.

In addition, a significant statistical dependency was observed both in AM and M between the single-subject scaling exponents and the sdIOI values. The DFA scaling exponents α correlated significantly and positively with the sdIOI in AM (Spearman ρ = 0.5330 in SEQ3 and ρ = 0.5966 in SEQ6; *p* < *p*th = 0.01). These outcomes associated in the pianists a larger degree of LRTCs with more variable timing performance, as has been reported previously for tapping or rhythm production experiments (Hennig et al., 2011; Smit et al., 2013). Note, however, that the statistical dependency was not significant in M. In addition, the power law scaling exponents β showed a trend of significance for an expected similar negative correlation with the sdIOI (range ρ = 0.45–0.50, *p* < 0.10).

### Influence of deep brain stimulation of the subthalamic nucleus on the temporal correlations in sequences of musical performance

**Figure 4:**
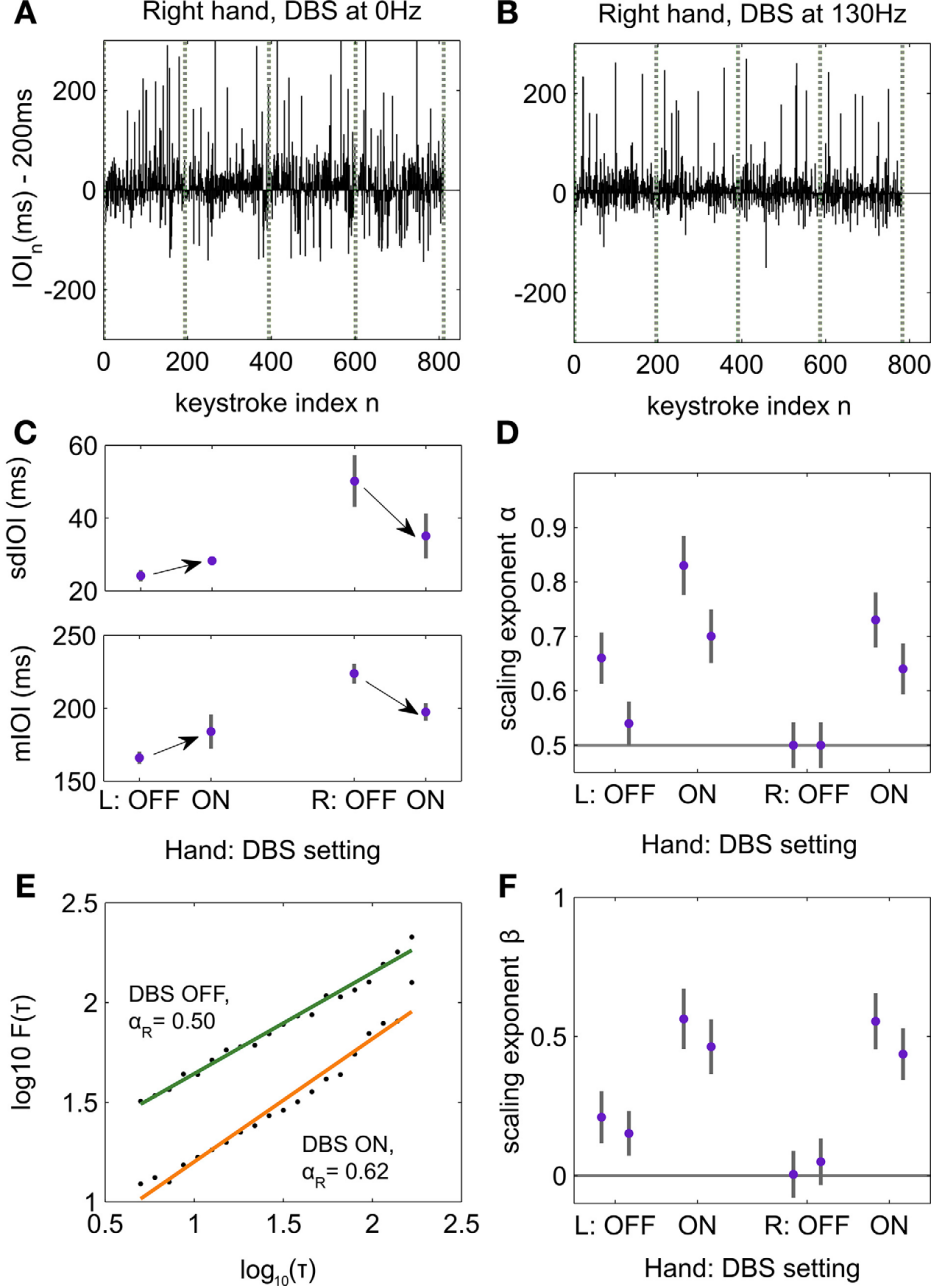
**Influence of deep brain stimulation (DBS) on the structure of temporal correlations during piano performance. (A)** Time series of deviations in inter-onset-interval (IOI, ms) values during performance with the tremor-affected right hand and without DBS. Vertical lines denote boundaries between trials corresponding to renditions of the same sequence type. The IOI fluctuates around the nominal IOI of 200 ms. **(B)** Same for performance with the tremor-affected hand during DBS ON (clinical settings at 130 Hz). **(C)** Average and SE (gray lines) values for mIOI and timing accuracy (sdIOI) are displayed for each hand and DBS setting separately. Assessment of the sdIOI and mIOI across trials with a two-factorial design with factors hand (L/R) and DBS (ON, OFF) demonstrated a significant interaction (*p* = 0.0001). Hence, whereas DBS ON improved timing parameters in the affected hand, it deteriorated timing performance in the less affected hand. Arrows denote the significant changes between ON and OFF DBS for each timing measure as assessed in a *post-hoc* analysis with pairwise paired permutation tests for each factor separately (Right hand: *p* = 0.0078 for changes in mIOI and sdIOI; Left hand: *p* = 0.03 for changes in mIOI, trend of significance for changes in sdIOI, *p* = 0.08). **(D)** First-order DFA scaling exponents α for each hand and DBS setting, separately for each of the two recorded conditions with DBS ON, and two recorded conditions with DBS OFF. The gray bars accompanying each value indicate the intrinsic error in the estimation of scaling exponents with DFA. DBS OFF led to uncorrelated fluctuations for the tremor-dominant hand (α ~ 0.5). In the less affected left hand DBS ON enhanced the degree of LRTC exhibited in the time series of temporal fluctuations. Note the replicability of the scaling exponents across the two conditions of each kind. **(E)** Log–log plot of the fluctuation function *F* (τ) against increasing time scales τ spanning 5–200 keystrokes corresponding with performance of the tremor-dominant right hand. Under DBS ON, DFA demonstrated power-law scaling behavior in the time series with scaling exponents in the LRTC range (0.5 < α < 1; data fitted to an orange line). By contrast, without DBS performance with the affected hand was characterized by uncorrelated fluctuations (α ~ 0.5, data fitted to the green line). The scaling exponents α are extracted from the slope of the linear fit. **(F)** PSD scaling exponents β and corresponding intrinsic error bars for each hand and DBS setting, as in **(D)**. We replicated the findings obtained with DFA, such that DBS OFF induced uncorrelated temporal fluctuations (β ~ 0) in the right hand, and reinstated power law 1/*f*^β^ behavior under DBS ON.

In each stimulation condition, the sdIOI and mIOI of both sequence types were not significantly different across the musical rendition or between the first and last trial of that sequence type (*p* > *p*th = 0, with single *p* > 0.05 in all cases). Hence, we concatenated the four renditions of each sequence type to generate the time series of IOIs for performance with the left and right hands, separately, and for each stimulation condition (Figures 4A,B). As expected, timing parameters across all stimulation conditions were different for the left and right hand, with faster and more regular timing in the less affected left hand [paired permutation test across stimulation conditions: sdIOI 24.7 (0.48) ms for left hand and 42.6 (2.69) ms for right hand, *p* = 0.001, *PS*_dep_ = 0.8125; mIOI 173.6 (10.24) ms and 210.7 (9.77) ms for left and right hand, respectively, *p* = 0.002, *PS*_dep_ = 0.7500]. These outcomes also emphasize that the patient performed at a faster tempo than the nominal 200 ms IOI. He reported that 200 ms IOI felt two slow for performing with his less affected left hand and therefore he willingly performed at a faster tempo.

A two-factorial analysis of the timing parameters with factors hand (L/R) and DBS OFF/ON (clinical settings) demonstrated a significant interaction (*p* = 0.0001 for sdIOI and mIOI, synchronized rearrangements across trials). This result suggested that DBS had a different effect on the timing performance of either hand. A *post-hoc* pairwise comparison between DBS ON and OFF for the less affected hand demonstrated a significant increase in tempo (*p* = 0.03, *PS*_dep_ = 0.7500) and a trend of significance for an increased timing unevenness under DBS ON (*p* = 0.08, PS_dep_ = 0.7500; Figure 4C). By contrast, DBS OFF compared to ON was associated with slower tempo and poorer temporal accuracy in the tremor-affected right hand (*p* = 0.0048, *PS*_dep_ = 1 in both cases; Figure 4C). This result established that during DBS the tremor severity decreased leading to an improved fine motor control in the right hand. Thus, whereas DBS ON consistently improved timing performance in the tremor-affected hand, it impaired tempo and timing regularity in the less affected hand.

In the time series of IOI corresponding to piano performance with the less affected left hand first-order DFA demonstrated across the four DBS settings (ON, OFF, ON, OFF) the presence of LRTCs spanning a range of 5–200 keystrokes (Figures 4D,E). The scaling exponents α were all in the LRTC range: 0.54–0.83 [mean 0.68 (0.059), exponents significantly above 0.5, *p* = 0.002, *PS*_dep_ = 1], with larger exponents in the DBS ON condition. The sequential structure of interval production with the tremor-dominant hand exhibited LRTC exclusively during DBS ON with scaling exponents 0.64 and 0.73. By contrast, the temporal intervals were uncorrelated with DBS OFF (α ~ 0.5). Consequently, the effect of subthalamic DBS contralateral to the tremor-dominant hand was to induce persistent long-range correlations in the dynamics of temporal intervals during piano performance. Similar outcomes were obtained when assessing by means of PDF the 1/*f*^β^ power-law scaling behavior in the time series from the different DBS settings (Figure 4F). Note that all reported scaling exponents were corrected to compensate for the bias induced by DFA and PSD (see Section Materials and Methods). The intrinsic errors in the estimation of the DFA scaling exponents were in the range 0.02–0.04. In the estimation of the PSD β exponents the intrinsic errors were in the range 0.07–0.11.

In sum, the general effect of DBS in performance was two-fold: (1) it improved timing performance in the tremor-affected hand and correspondingly shifted the DFA scaling exponents from the regime of uncorrelated noise to the LRTC range. (ii) In the less affected hand the scaling exponents increased in parallel to the transition DBS OFF → ON, yet within the LRTC range. In addition, timing performance was more compromised under DBS ON. As in the previous study, larger scaling exponents *within* the LRTC range were associated with poorer temporal accuracy.

## Discussion

The present work investigated in two different studies the correlational structure of the fluctuations in the IOI during skilled piano performances. We focused on the assessment of the slower trends of variation in the production of temporal intervals (drift), which have been related to short-term memory processes (Staddon, 2005). Unlike drift, interval-by-interval variability is typically random or anticorrelated and has been associated with the motor system (Gilden et al., 1995).

In both our studies, participants performed music pieces from memory. The pieces consisted of isochronous IOIs and note durations and had been intensively rehearsed at an instructed tempo. In the first study we specifically assessed the influence of auditory feedback on the correlational structure of the fluctuations of IOI during the musical performances. Exclusively musical sequences that fulfilled the criterion of showing no significant change of average tempo and unevenness of IOI (sdIOI) between the beginning, middle and end of each musical rendition or between the first and last rendition were selected. For those selected pieces, different renditions of the same piece were concatenated to enable analysis of longer time series by means of DFA and spectral power analysis (Peng et al., 1995; Pilgram and Kaplan, 1998).

The main findings were the presence of long-range (persistent) correlations in the IOI fluctuations extending from 5 up to 200 keystrokes (the length of each rendition) both when playing with or without auditory feedback. Similarly, LRTC in continuation tapping and in sensorimotor synchronization have been shown to be independent of auditory feedback (Chen et al., 2002), and to be stable after training (Madison et al., 2013). Scaling exponents were in the LRTC range (0.5 < α < 1 or 0 < β < 1) for most pianists (between 12/17 and 15/17 depending on the condition and musical sequence). This outcome demonstrated that the unintended temporal deviations from a perfectly regular (isochronous) tempo that manifest in skilled performance over several time scales are not uncorrelated but rather share long-term dependency. Therefore, there is not a characteristic temporal scale that dominates the fluctuation function. The degree of LRTC was more pronounced (larger α and β exponents) in pianists playing with larger standard deviation of IOI, in line with previous studies of finger tapping and rhythm production (Hennig et al., 2011; Smit et al., 2013). The scaling exponents obtained for the different musical sequences 3 and 6 were not correlated across pianists, which suggests that the degree of LRTC was expressed differently in each pianist depending on the musical structure. Supporting this interpretation, the correlational structure of pitch and rhythm of notated musical compositions also exhibits characteristic LRTC but with wide variability within and across composers (Voss and Clarke, 1978; Levitin et al., 2012).

The structure of the temporal correlations in skilled piano performance resembles the presence of 1/*f*^β^ power-laws described in a wide variety of behavioral tasks such as self-paced isochronous finger tapping, production of a rhythmic pattern synchronized to a metronome, walking, circle drawing, or audiovisual threshold-stimulus detection (Gilden et al., 1995; Chen et al., 1997, 2002; Torre and Delignières, 2008; Delignières and Torre, 2009; Hennig et al., 2011; Palva et al., 2013). Several models have been put forward to account for the manifestation of LRTC in behavior (Gilden et al., 1995; Chen et al., 1997; Torre and Delignières, 2008; Delignières et al., 2009). These models partly build upon existing accounts of self-paced or synchronized tapping in which the sources of variability in the generation of inter-tap intervals are considered to be uncorrelated or anticorrelated (e.g., Wing and Kristofferson, 1973; Vorberg and Wing, 1996, see review in Repp and Su, 2013). The abovementioned influential model of Gilden et al. (1995) for self-paced production of temporal or spatial intervals proposed that an internal timekeeper prescribing the intervals to the motor system is a source of 1/*f*^β^ noise. Along a similar line, modeling a timekeeper as source of 1/*f*^β^ noise can account for the long-range dependency of the asynchronies (*synchronization errors*) generated during tapping to an external rhythm or metronome (Delignières et al., 2008, 2009; Torre and Delignières, 2008).

Playing a music melody from memory does not exclusively involve the temporal organization of a long sequence of events. Music performance relies on the interplay between several cognitive processes such as the retrieval from memory of the musical structure and pitch content, the preparation in advance of the events planned for production and, last but not least, the communication of expressive effects (Palmer, 1997; Janata and Grafton, 2003; Zatorre et al., 2007; Hallam et al., 2008). These processes likely add additional temporal variability to the performance, as reflected in the automatic slowing following a pitch error or during the conflicting co-representation of pitch elements prior to production, and in the intentional expressive timing effects (Palmer, 1997; Palmer and Pfordresher, 2003; Herrojo Ruiz et al., 2009, 2011; Maidhof et al., 2009). The presence of LRTC in piano performance thus suggests that the generation of 1/*f*^β^ noise cannot be exclusively attributed to a cognitive “timekeeper” system issuing a timing motor command. A very influential hypothesis in this context is that the manifestation of power-law scaling behavior is an indicator of self-organized criticality (Bak et al., 1988; Jensen, 1998). Criticality in statistical physics corresponds with the property of a system with spatial and temporal degrees of freedom at the point of undergoing a second order phase-transition between an ordered state and a random state (Essam et al., 1972). Close to and at the critical point the susceptibility of the system is maximal, thereby enabling the emergence of a large variety of spatiotemporal patterns that are metastable (i.e., persist for an extended period of time, yet are not at equilibrium) and exhibit slowly decaying spatiotemporal correlations. The concept of self-organized criticality was expanded to dynamical systems to account for the case of an internal dynamics driving the system to its critical point (Bak et al., 1987, 1988). It follows that the presence of LRTC and 1/*f*^β^ laws in behavioral performance can be considered to be brought about by the brain's complex spatiotemporal dynamics operating close to criticality (Kelso et al., 1992; Linkenkaer-Hansen et al., 2001; Chialvo, 2010). This view is supported by recent data showing that behavioral scaling exponents during synchronization tapping and perceptual tasks correlate across subjects with the scaling exponents of LRTC exhibited in the neuronal dynamics of *task-related* regions (Palva et al., 2013; Smit et al., 2013). Similarly, the manifestation of 1/*f*^β^ noise in the production of temporal intervals during music performance could emerge from the complex spatiotemporal dynamics of the multiple neural systems engaged in the task. These systems include, but are not limited to, primary and secondary motor areas, auditory areas, cingulate cortex, basal ganglia, and cerebellum (Altenmüller et al., 2006; Zatorre et al., 2007; Hallam et al., 2008).

Our second study provided a window into the previous hypothesis by investigating how altering the brain dynamics along cortico-basal ganglia loops using subthalamic DBS may affect behavioral scaling exponents during skilled piano performance. In our patient, the analysis of the performance with the less affected hand replicated the findings of the first study related to the presence of LRTC in the temporal fluctuations during piano performance. The regime of scale-invariant power-law behavior observed in the temporal fluctuations spanned 5 up to 200 keystrokes and a low frequency range (<0.16 Hz). Interestingly, the DFA and PSD scaling exponents increased within the LRTC regime during DBS as compared to performance when DBS was switched off. This change was paralleled by a shift to a poorer timing performance during DBS, which underscores the association found in the first study between larger scaling exponents within the range of LRTC and poorer temporal performance. Note that a relative deterioration in motor symptoms and behavioral output in the less affected side during bilateral DBS has been previously reported in PD patients with prominent asymetric motor symptoms (Johnsen et al., 2009). In contrast, timing performance with the right tremor-affected hand was improved during DBS compared to the OFF condition similar to the improvement in clinical motor symptoms as assessed by UPDRS-III score. In addition, DFA and PSD analysis demonstrated that OFF DBS the temporal fluctuations were uncorrelated (α ~ 0.5 and β ~ 0). The LRTC regime in the tremor-affected hand was, however, reinstated by DBS at clinically effective parameters. The outcomes in the tremor-affected hand are consistent with evidence from non-invasive auditory rhythmic stimulation in PD for a restauration of the 1/*f*^β^ noise in gait of these patients (Hove et al., 2012), a property characteristic of healthy human gait (Hausdorff, 2009). In addition, at the neuronal level, the temporal dynamics in the basal ganglia of PD patients exhibit a larger degree of long-range dependency following pharmacological treatment with apomorphine (Cruz et al., 2009) or levodopa (Hohlefeld et al., 2012). Thus, investigations in PD patients emphasize a typical disruption of 1/*f*^β^ noise and long-range correlations at the behavioral and neuronal levels when the patients are in their more compromised clinical state (OFF medication, OFF DBS). Our study significantly demonstrates that the treatment with STN-DBS in a PD patient with asymmetric motor symptoms successfully restores in the most affected tremor-dominant hand the power-law scaling behavior in the temporal fluctuations during skilled piano performance. Presumably, alteration via DBS of the pathological network dynamics along the cortico-basal ganglia-thalamocortical loops might induce a larger degree of task-related and frequency-specific LRTC at the neural level (Hohlefeld et al., 2013). Accordingly, LRTC in the behavioral time series might appear as a macroscopic manifestation of restored dynamics and enhanced neuronal LRTC along cortico-basal ganglia circuits. However, future work investigating in parallel the effects of DBS on the structure of the correlations in behavior and cortical-subcortical activity should clarify this issue.

More generally, the effect of clinically effective STN-DBS on average timing parameters during tapping tasks is to improve the production of temporal intervals (Chen et al., 2007; Eusebio et al., 2008). In the specific case of violin performance, the effect of unilateral STN-DBS on the *intended* expressive timing patterns was recently assessed (Van Vugt et al., 2013). This study found an improved musical expression and timing performance during STN-DBS, yet the latter exclusively when performing in parallel with a metronome. The outcomes, however, might be specific to the treatment of unilateral DBS and might not directly relate to our findings with bilateral DBS.

An important difference between music performance with or without metronome is that in the first case production of temporal intervals is externally paced, whereas in the second case it is primarily internally generated. The basal ganglia seem to be important in both processes, with a predominant involvement of the putamen in the prediction and continuation of the beat and a role of the caudate and ventral striatum in prediction error during externally paced beat detection (Grahn and Brett, 2008; Grahn and Rowe, 2009; Schiffer and Schubotz, 2011). Considering the previous evidence, an interesting direction of future research would be to investigate how subthalamic DBS applied at different contacts of the DBS electrodes within either limbic, associative or motor STN territories would affect differently the temporal fluctuations in performance with or without metronome.

## Funding

This research is supported by the German Research Foundation (DFG) through projects HE 6103/1-1 and HE 6013/1-2 to María Herrojo Ruiz, and project HE 6312/1-2 to Holger Hennig.

### Conflict of interest statement

The authors declare that the research was conducted in the absence of any commercial or financial relationships that could be construed as a potential conflict of interest.
