# Supplementary material for: Long-range correlation properties in timing of skilled piano performance: the influence of auditory feedback and deep brain stimulation
Source: Front Psychol. 2014 Sep 25;5:1030. doi: 10.3389/fpsyg.2014.01030 (PMC4174744; doi:10.3389/fpsyg.2014.01030)
Supplement: Supplementary file 1 [file Image1.PDF]

Supplementary Figure S1

(A) Example of a realization of fractional Brownian motion of length N = 2000 with known Hurst exponent (H = 0.55). 500 realizations of a fBn process were generated with MATLAB® function wfbm for each true known Hurst exponent in the range 0.5: 0.1: 1. **(B)** Illustration of a realization of a fractional Gaussian motion process (fGm) generated as the increment process (differentiation) of the fBm time series in **(A)** and with the same Hurst exponent. Estimation of the scaling exponents by means of PSD (exponent β_m, with T_0 = 1s) and DFA (α_m) was then made in each of the 500 realizations of the fGm process of known Hurst exponents. **(C)** Bias (mean[α_m] – H) in the estimation of the scaling exponent with true value α = H for realizations of continuous time series of length N = 2000 and (gray lines) and for time series consisting of 10 concatenated time series of length M= 200 as realizations of an fGn process (total length N = 2000, red line).
(D) Intrinsic error (std[α_m]) in the estimation with DFA. Color code as in **(C)**. **(E)** Bias (mean[β_m] – [2H-1]) in the estimation of the scaling exponent β, which has a true value β = 2H-1 in the case of stationary time series. Color code as in **(C)**. Intrinsic error (std[β_m]) in the estimation with PSD.

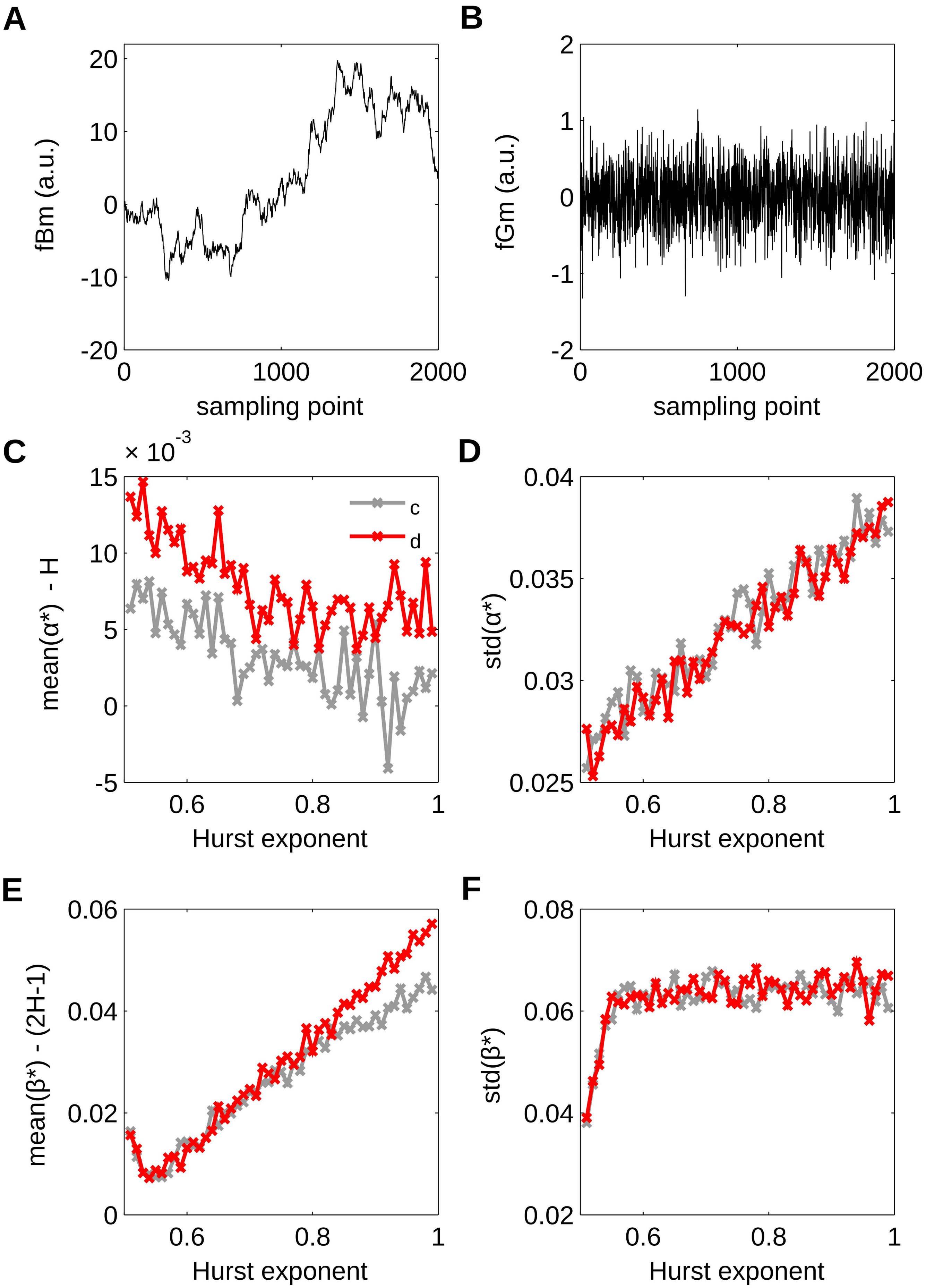
A
fBm (a.u.)
sampling point
B
fGm (a.u.)
sampling point
C
× 10^-3
mean(α*) - H
c
d
Hurst exponent
D
std(α*)
Hurst exponent
E
mean(β*) - (2H-1)
Hurst exponent
F
std(β*)
Hurst exponent
